# Characterizing coagulation responses in humans and nonhuman primates following kidney xenotransplantation—A narrative review

**DOI:** 10.1002/ajh.27506

**Published:** 2024-10-15

**Authors:** Ali Zidan, Adham H. El‐Sherbini, Abdelrahman Noureldin, David K. C. Cooper, Maha Othman

**Affiliations:** ^1^ Faculty of Medicine University of Toronto Toronto Ontario Canada; ^2^ Faculty of Medicine University of Ottawa Ottawa Ontario Canada; ^3^ Center for Transplantation Sciences, Department of Surgery Massachusetts General Hospital, Harvard Medical School Boston Massachusetts USA; ^4^ Department of Biomedical and Molecular Sciences, School of Medicine Queen's University Kingston Ontario Canada; ^5^ School of Baccalaureate Nursing St. Lawrence College Kingston Ontario Canada; ^6^ Clinical Pathology Department, Faculty of Medicine Mansoura University Mansoura Egypt

## Abstract

The recent report of the first pig kidney transplant in a living human brings hope to thousands of people with end‐stage kidney failure. The scientific community views this early success with caution as kidney xenotransplantation exhibits many challenges and barriers. One of these is coagulation dysregulation. This includes (i) pig von Willebrand Factor (vWF) interaction with human platelets, which can induce abnormal clotting responses, heightening the risk of graft failure, (ii) the inefficiency of pig thrombomodulin in activating human protein C, which emphasizes the species‐specific variations that aggravate coagulation challenges, and (iii) the development of thrombotic microangiopathy in the pig grafts and the occurrence of systemic consumptive coagulopathy in the recipients. Indeed, coagulation dysregulation largely results from differences in endothelial cell response and incompatibilities between pig and human coagulation–anticoagulation pathways. These barriers can be resolved by modifications to pig vWF and the expression of human thrombomodulin and endothelial protein C receptors in pig cells, serving as strategic interventions to align the coagulation systems of the two species more closely. These coagulation challenges have clinical implications in how they affect graft survival and patient outcome. Genetic engineering of the organ‐source pig and the administration of various drugs have assisted in correcting this coagulation dysregulation. Hence, comprehending and controlling coagulation dysregulation is crucial for progress in xenotransplantation as a viable option for treating patients with terminal kidney disease.

**Abbreviations**
aPC: activated protein C
CC: consumptive coagulopathy
EC: endothelial cells
EPCR: endothelial protein C receptor
NHP: nonhuman primate
TF: tissue factor
TFPI: tissue factor pathway inhibitor
TMA: thrombotic microangiopathy
vWF: von Willebrand factor

## 1 GLOBAL KIDNEY DISEASE AND XENOTRANSPLANTATION

Kidney disease, affecting over 850 million people globally, is anticipated to become the 5th leading cause of death by 2040.^1^ In the United States (US), there are more than 89 000 patients on the kidney transplant waiting list as of May 2024, with approximately more than 25 000 kidney transplants performed annually.^2^ The demand far exceeds the supply, as evidenced by the daily death of 17 patients on the waiting list and an annual addition of approximately 34 000 new patients in the US.^3^ Current statistics indicate that around 40% of patients on the waitlist are unlikely ever to receive an allograft.^4^

This shortfall has shifted research focus towards kidney xenotransplantation (cross‐species transplantation) as a viable solution.^5^ This process, with the goal of transplanting a pig kidney into a human, may unlock an essentially unlimited organ supply and overcome blood type compatibility issues.^6^ Pigs as sources of kidneys are preferred over nonhuman primates (NHPs) for a number of reasons^6^ (Table 1).

**TABLE 1:** The advantages and disadvantages of the pig as a potential source of organs and cells for humans.

Availability	Unlimited, whenever required
Breeding potential	Good
Period to reproductive maturity	4–8 months
Length of pregnancy	114 ± 2 days
Number of offspring	5–12
Growth	Rapid (adult human size within 6 months)^a^
Size of organs for all ages of humans	Adequate
Anatomical similarity to humans	Close
Physiological similarity to humans	Close
Relationship of immune system to humans	Distant
Knowledge of tissue typing	Considerable (in selected herds)
Blood type compatibility with humans	ABO‐blood type compatibility can be assured (All pigs will be of type O [non‐A])
Experience with genetic engineering	Considerable
Risk of transfer of infection (xenozoonosis)	Low
Availability of designated pathogen‐free pigs	Yes
Cost of maintenance	Under the biosecure designated pathogen‐free conditions required by the national regulatory authorities, the costs will be significant.
Public opinion	Generally supportive

^a^ Various miniature pigs reach a maximum weight of 10%–50% of the weight of domestic pigs.

However, significant challenges remain, such as high rates of antibody‐mediated rejection (estimated to be 42% after xenotransplantation vs. 6% after allotransplantation), acute cellular rejection, physiological incompatibilities, and the risk of cross‐species infection.^7^ These various reactions can contribute to abnormal coagulation responses which collectively (through their interconnected nature) may combine to cause graft destruction. Coagulation dysfunction can take the form of thrombosis or bleeding, platelet sequestration, reduced fibrinogen, increased D‐dimer, and prolonged clotting time. Notably, studies in NHPs have resulted in thrombotic microangiopathy (TMA) in the pig graft and consumptive coagulopathy (CC) in the NHP recipient.^8^, ^9^

Therefore, understanding and managing coagulation dysregulation is essential for advancing xenotransplantation as a sustainable treatment for end‐stage kidney disease.^9^ The human or NHP response to a pig kidney graft is complex, involving immune and inflammatory responses (neither of which will be reviewed in detail here), and physiological incompatibilities, including coagulation dysfunction.

During the preparation of this review, the first pig kidney transplant in a 62‐year‐old Richard Slayman and a second in 54‐year‐old Lisa Pisano were announced. Although the former has since passed away and the latter has had the pig kidney removed, and has also passed away, their sacrifices for the medical community enable better understanding of the complex pathologies behind the unsuccessful attempts and remain a promise to thousands of patients with end‐stage kidney disease across the globe.^10^ This review will provide a timely update on the current knowledge and challenges associated with coagulation dysregulation while we continue to watch with optimism the success of the first trial.

## 2 NORMAL COAGULATION AND ITS COMPLEX REGULATORY MECHANISMS

Coagulation is a two‐phase process comprising primary and secondary hemostasis.^11^ In primary hemostasis, vasoconstriction initially reduces blood loss, followed by the formation of a platelet plug.^11^ This process is initiated by the release of von Willebrand Factor (vWF) from endothelial cells (ECs), which binds to collagen in the exposed subendothelium, and interacting with platelet s via platelet glycoprotein GPIba leading to tethering of platelets to the injured endothelium; platelet adhesion, activation, and finally aggregation via platelet glycoprotein IIB/IIIa which is also a VWF receptor, eventually forming a plug.^11^ Primary hemostasis is regulated by a number of inhibitory mechanisms like endothelial nitric oxide, and prostacyclin and CD39 (an extracellular ATPase).^11^

**FIGURE 1:**
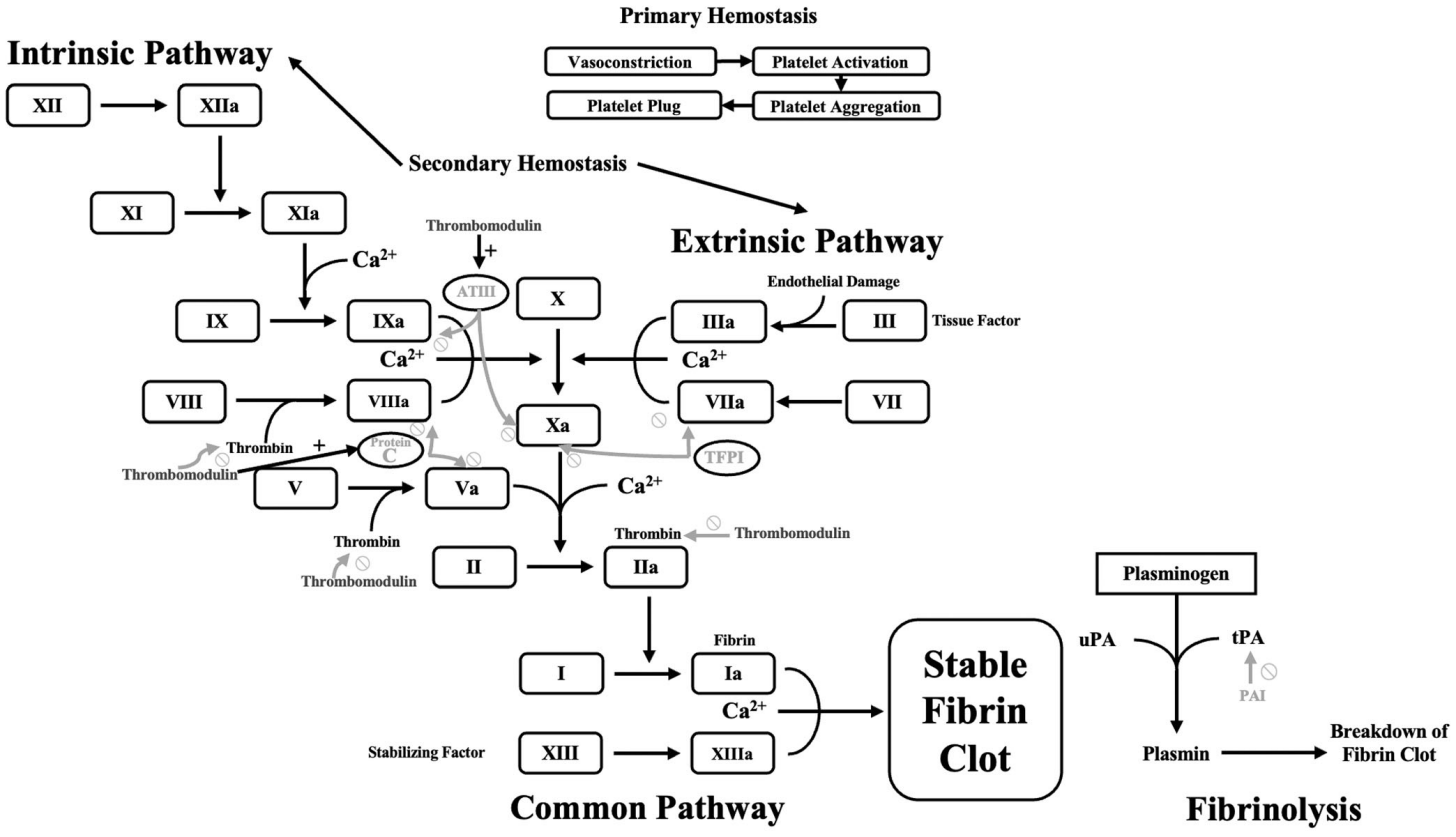
Overview of coagulation and fibrinolytic systems, basic regulatory mechanisms including primary and secondary hemostasis. These mechanisms are functionally similar in both humans and NHP. Including the procoagulant intrinsic, extrinsic, and common pathways and clot dissolving fibrinolytic pathway. AT‐III, antithrombin‐III; PAI, plasminogen activator inhibitor; TFPI, tissue factor pathway inhibitor; tPA, tissue plasminogen activator; uPA, urokinase‐type plasminogen activator.

Secondary hemostasis involves the formation and stabilization of a fibrin‐based hemostatic plug (Figure 1). It is characterized by the interactions of zymogens (inactive enzymes), scaffolding cofactors, and activated enzymes. Triggered by vessel injury and tissue factor (TF) release, the extrinsic pathway is activated first.^12^ Here, the activated enzyme (e.g., Factor [F] VIIa) binds TF to activate zymogen FX into FXa, catalyzing the conversion of prothrombin to thrombin.^12^ Most of the coagulation factors are synthesized by hepatocytes within the liver. Thrombin then cleaves soluble fibrinogen into fibrin, stabilized by FXIIIa into a crosslinked fibrin clot.^12^ The intrinsic pathway, also known as the amplification pathway, involves the intrinsic tenase complex (FIXa, FVIIIa, and FX), amplifying FXa production significantly.^12^

Regulation of coagulation is maintained by a group of natural inhibitors (Figure 1). Tissue factor pathway inhibitor (TFPI) is a Kunitz‐type serine protease inhibitor from ECs that inhibits the TF/VIIa complex.^12^, ^13^ It is regarded as the most important endogenous inhibitor of the extrinsic pathway of coagulation.^14^ Antithrombin, produced by hepatocytes, is another inhibitor that inactivates mainly thrombin and to less extent factors IXa, Xia, and XIIa in the tenase complex.^12^ Protein C, a zymogen activated by thrombomodulin (a membrane protein expressed on ECs, with high affinity binding to thrombin) forming the thrombin‐thrombomodulin complex, requires protein S as a cofactor and inactivates FVIIIa and FVa.^12^ Finally, fibrinolysis, mediated by plasminogen activators, uPA and tPA, converting plasminogen into plasmin, breaks down the fibrin clot (Figure 1).^15^ This process is controlled by plasminogen activator inhibitor (PAI), ensuring proper fibrinolysis balance.^15^

## 3 COAGULATION DYSREGULATION IN PIG KIDNEY XENOTRANSPLANTATION

**FIGURE 2:**
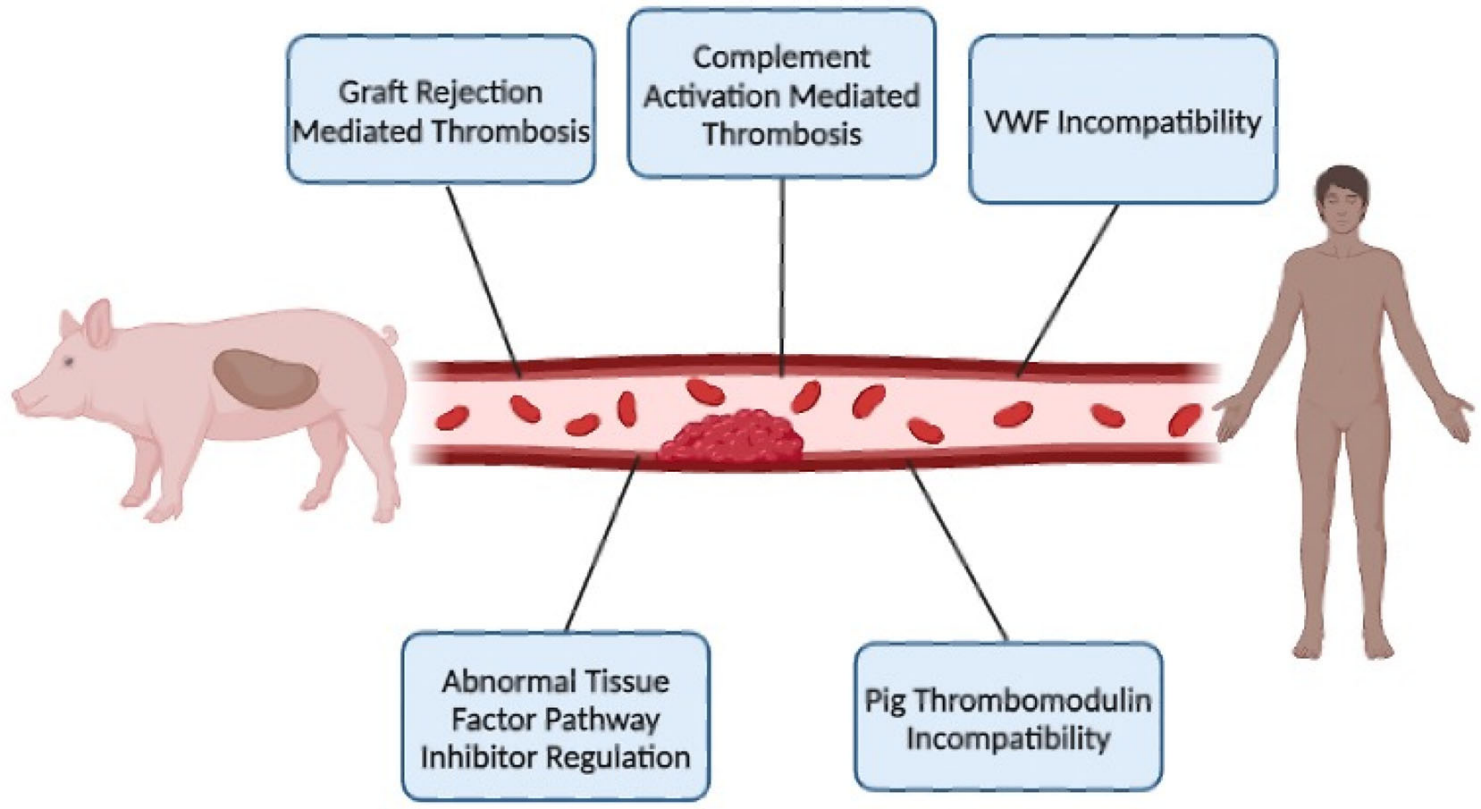
Proposed mechanisms of coagulation dysregulation in kidney xenotransplantation. The broad range of these mechanisms, including graft rejection, complement activation, and coagulation system incompatibilities and regulation, highlights the abundance of issues that may cause coagulation dysregulation in kidney xenotransplantation. Thus, failure to overcome these challenges may lead to undesirable outcomes during and after xenotransplantation. (Created with BioRender.com.). [Color figure can be viewed at wileyonlinelibrary.com]

There are several coagulation and anticoagulation processes that can be dysregulated during pig kidney xenotransplantation (Figure 2). For example, in its native state, pig vWF interacts abnormally with human platelets, resulting in spontaneous platelet activation and aggregation without the need for shearing force.^16^ This abnormality may induce clotting to the detriment of xenograft outcomes. In addition, in wild‐type (i.e., genetically‐unmodified) nontransplanted pig kidneys, staining for vWF is typically weak, suggesting that transplantation and the subsequent immune response contribute to EC activation.^17^ Controlling this activation is essential to avoid post‐transplant thrombotic complications.

A significant challenge in xenotransplantation is the inefficiency of pig thrombomodulin to activate human protein C.^18^ Pig thrombomodulin is structurally only approximately 69% similar to its human counterpart.^19^, ^20^ In recipients of pig xenografts, this incompatibility can cause both coagulation dysregulation and an increased inflammatory response (compared with human thrombomodulin). Ineffective interaction between porcine thrombomodulin and human thrombin leads to a state favoring coagulation.^9^ Moreover, Siegel et al. found that the loss of thrombomodulin from the surface of ECs during the progression of an inflammatory process displays functional incompatibility with xenograft acceptance.^21^ The transgenic introduction of human thrombomodulin into pig ECs is associated (when exposed to blood from baboons with pig organ xenografts) with local regulation of the inflammatory response and a reduction in the activation of the complement system.^22^ Exemplarily, oxidative inactivation of natural anti‐thrombotic proteins following high‐level expression of human thrombomodulin in pig xenografts has been implicated with the restoration of anticoagulant properties during xenograft rejection.^23^ Combining the transgenic insertion approach with immunosuppressive therapy has been successful in preventing immune rejection and coagulation problems, significantly extending the survival of pig heterotopic (i.e., non‐life‐supporting) heart grafts in baboons to >900 days.^24^ Intertwined with this, blocking inflammatory pathways (specifically NF‐κB signaling) has been shown to significantly upregulate pig thrombomodulin following pig organ xenotransplantation models.^22^

Pig ECs expressing human thrombomodulin have shown promise in inhibiting prothrombinase activity and impairing coagulation.^25^ Under physiological conditions, thrombomodulin and endothelial protein C receptor (EPCR) on ECs are crucial for inhibiting coagulation through activated protein C (aPC)‐mediated regulation.^26^, ^27^ From evaluating a variety of original observations, Cowan et al. concluded that the aPC‐generating capacity of pig thrombomodulin in the xenogeneic setting is <10% of that of human thrombomodulin.^9^ In human umbilical vein ECs, aPC is readily generated with human thrombin and protein C.^27^ However, this aPC production is not observed in porcine cells under the same conditions, indicating an incompatibility between human thrombin, protein C, and porcine thrombomodulin/EPCR.^27^ This leads to enhanced clotting in human blood, as evidenced by increased thrombin–antithrombin complex formation, likely due to the porcine cells' inability to generate aPC.^27^

## 4 MAJOR COAGULOPATHIES IN PIG KIDNEY XENOTRANSPLANTATION

### 4.1 Immune/inflammatory‐mediated concerns

Antibody‐mediated complement activation following pig kidney xenotransplantation can promote thrombin generation and platelet aggregation, further exacerbating coagulation dysregulation.^28^, ^29^ Porcine cells express three primary glycan antigens (α‐Gal, Neu5Gc, and Sda) which are the products of specific glycan synthesis genes (Table 2).^30^ In humans, the genes corresponding to α‐Gal and Neu5Gc have become pseudogenes, and thus, these antigens are absent, exacerbating the immune response when exposed to porcine cells.^31^ Wild‐type porcine kidney ECs exhibit high levels of binding by human immunoglobulins IgG and IgM, indicative of a strong potential for initiating immune responses upon transplantation into human hosts.^27^

**TABLE 2:** Known carbohydrate xenoantigens expressed on pig cells.

Carbohydrate (Abbreviation)	Responsible enzyme	Gene‐knockout pig
1. Galactose‐α1,3‐galactose (Gal)	α1,3‐Galactosyltransferase	GTKO
2. N‐Glycolylneuraminic acid (Neu5Gc)	CMAH	CMAH‐KO
3. Sda	β‐1,4 N‐Acetylgalactosaminyltransferase	β4GalNT2‐KO

Abbreviations: CMAH, cytidine monophosphate‐N‐acetylneuraminic acid hydroxylase (CMAH); CMAH‐KO, cytidine monophosphate‐N‐acetylneuraminic acid hydroxylase knockout (CMAH‐KO); GTKO, α1,3‐galactosyltransferase gene‐knockout (GTKO); β4GalNT2‐KO, β‐1,4 N‐acetylgalactosaminyltransferase knockout (β4GalNT2‐KO).

However, human serum antibody binding is significantly reduced to gene‐edited kidney ECs in which the expression of the three known pig glycan xenoantigens has been deleted (triple‐knockout [TKO] pig kidney ECs).^27^ Despite this reduction, in many human subjects, residual serum antibody binding, particularly of IgM, may persist.^27^ The binding of these antibodies initiates complement activation, leading to the production of surface‐bound C3b and soluble C3a.^27^ In contrast to human umbilical vein ECs, which show no C3b deposition when incubated with human serum, wild‐type pig kidney ECs demonstrate significant C3b deposition under identical conditions.^27^ This indicates that porcine complement‐regulatory proteins are less effective at mitigating human complement activation.^17^, ^27^, ^32^ Timely reviews of both complement and antibody‐related complications of xenotransplantation have been provided by Anand et al. and Cooper & Pierson.^5^, ^27^

### 4.2 Thrombotic microangiopathy (TMA)

Dysfunction of coagulation is exhibited by the development of thrombotic microangiopathy (TMA) in the pig grafts and the occurrence of systemic consumptive coagulopathy (CC) in the recipients. TMA may be triggered by a low‐grade immune response, infection, drugs, or procedures such as biopsies, and leads to graft damage.^9^ There is evidence that TMA is potentially influenced by TF‐independent mechanisms.^9^ Xenogeneic insult can activate endothelium and trigger subendothelial TF release, and this can be independent of the xenoreactive immune response. TF expression on monocytes and platelets can be induced by xenogeneic interactions with endothelium,^8^, ^9^ which results in the production of thrombin.^8^ Coagulation problems are also exacerbated by FGL2's direct conversion of human prothrombin to thrombin, which occurs without the need for TF.^33^ Additionally, inorganic polyphosphate (polyP), which is released by activated platelets, directly initiates the intrinsic coagulation pathway and causes the production of thrombin and fibrin.^9^, ^34^ Inflammation may also result in decreased expression and stability of EPCR and thrombomodulin.^9^, ^35^ The aforementioned mechanisms thus lead to tubulitis, glomerulitis, arteritis, peritubular capillaritis, interstitial inflammation, appreciable C4d deposition in the peritubular capillaries, and eventually cortical necrosis or interstitial hemorrhage.^36^

### 4.3 Consumptive coagulopathy (CC)

CC is characterized by reduced fibrinogen, increased D‐dimer, increased thrombin–antithrombin levels, and prolonged clotting time, potentially reflecting divergent transcriptional responses.^9^, ^37^ α1,3‐galactosyltransferase gene‐knockout (GTKO) pig kidneys (in which expression of the GaI antigen has been deleted, Table 2) can still activate the vascular ECs via complement or antibody pathways, resulting in CC.^9^, ^38^ Even low levels of anti‐nonGaI antibodies are associated with the loss of the graft over days, weeks, or months, from the development of TMA or CC.^6^ However, the expression of hEPCR may protect a pig kidney xenograft from developing CC, suggesting transgenic hEPCR overexpression as a means of avoiding coagulation dysregulation in kidney xenotransplantation.^37^, ^39^ Life‐supporting pig renal xenotransplantation in NHPs has extended recipient survival up to 499 days in the absence of CC, and the grafts maintained normal function.^40^ In addition, the administration of certain antiplatelet or systemic anticoagulant drugs may reduce the high risk of CC following kidney xenotransplantation.^41^

Thrombomodulin, which has an anticoagulant effect, may not be sufficient to counteract procoagulant factors during kidney xenotransplantation, resulting in CC.^42^ In some studies, the source of TF was presumed to be porcine in origin and implicated as the initiator of thrombosis and CC.^43^ Determining the origin of the TF that initiates the development of CC is essential to adequately characterize and prevent this complication in future NHP and human models.

## 5 GENETIC ENGINEERING AS A TOOL FOR OVERCOMING COAGULATION DYSREGULATION

Genetic engineering can help overcome graft rejection. Organs from GTKO pigs are associated with a decreased probability of hyperacute rejection.^5^ By reducing the number of antigenic targets, the risk of coagulation‐related complications may also be decreased.^9^ For the later phases of graft rejection, additional deletion of expression of the Neu5Gc and SDa antigens (produced by the CMAH and β4GalNT2 genes, respectively) (Table 2) has reduced the possibility of endothelial activation and subsequent clot formation.^44^ Selected genetically modified xenografts are summarized in Table 3.

**TABLE 3:** Common xenotransplantation kidneys previously utilized for studies in brain‐dead humans (Revivicor) and living persons (eGenesis).

Product name (if applicable)‐company	Human gene insertions	Knock outs for glycan antigens	Inactivated porcine endogenous retrovirus/other
EGEN‐2784‐eGenesis^27^	7 CD46, CD55, TBM, EPCR, CD47, TNFAIP3, HO1.	3 α‐Gal, Neu5Gc, Sd(a)	59 inactivated copies of PERV elements
UKidney™‐Revivicor^71^	6 CD46, CD55, TBM, EPCR, CD47, HO1	3 α‐Gal, Neu5Gc, Sd(a)	1 Pig GHR
GalSafe™‐Revivicor^71^	N/A	1 α‐Gal	N/A

Abbreviations: EPCR, endothelial cell protein C receptor; GHR, growth hormone receptor; HO1, heme‐oxygenase‐1; Neu5Gc, N‐glycolylneuraminic acid; PERV, porcine endogenous retroviruses; TBM, thrombomodulin; TNFAIP3, tumor necrosis factor alpha‐induced protein 3; α‐Gal, galactose‐α‐1,3‐galactose.^27^, ^71^

Deletion of expression of all three known carbohydrate xenoantigens (TKO pigs) (Table 2) is likely to be the foundation for the first clinical trials, particularly given its potential in reducing antibody binding to the graft, which reduces complement activation and subsequently reduces coagulation dysfunction.^27^ However, more specific gene‐editing directed to the coagulation incompatibilities is more effective in preventing TMA and CC.

### 5.1 vWF‐deficient pigs or pigs with humanized vWF

Altering anticoagulant genes has been a source of overcoming coagulation dysregulation. One of the key areas has been vWF. vWF‐deficient genetically engineered pigs have been produced, which has marginally improved the survival of pig‐to‐NHP lung xenotransplantation (an extremely difficult model).^40^, ^45^ However, this modification alone has not been sufficient in kidney xenotransplantation, indicating the complex nature of immune responses on different organ systems.^29^ Thus, the idea of humanizing pig vWF was considered.^29^

This entails substituting human sequences for the particular exons of the pig vWF gene that are involved in binding to glycoprotein Ib (GPIb) on platelets.^29^ Both human and pig vWF contain the A1 domain within the amino acid 449–728 range, crucial for binding to GPIb receptors on platelets. This domain forms an intrachain loop facilitated by disulfide linkages (cys509 to cys695), which is integral for its function.^46^ However, differences in how this domain interacts with the human GPIb receptor lead to distinct outcomes. Human vWF (hvWF) in its recombinant soluble form (hvWF‐A1) binds to GPIb but does not initiate platelet aggregation without the presence of multimeric forms. By contrast, porcine vWF, even in the absence of ristocetin or shear stress, can aggregate and activate human platelets through GPIb interactions.^46^ Thus, the purpose of this alteration is to lessen the pig vWF‐induced spontaneous aggregation of human platelets.^29^

Studies have suggested that transgenic expression of humanized vWF may be able to alleviate coagulation disorders in pig xenograft recipients.^16^ It is still unclear exactly how this alteration will affect the long‐term survival of a xenograft.^16^, ^29^ Moreover, brain‐dead humans (a model previously used for kidney xenotransplantation) have been associated with imbalanced ADAMTS13(a vWF‐cleaving protease), which can lead to microthrombi formation in donor organs.^11^ Understanding vWF compatibility in xenotransplantation is further complicated by potential differences in how humans and NHP interact with porcine vWF, considering that a species‐dependent variability in ADAMTS13‐mediated proteolysis of human recombinant von Willebrand factor exists.^11^ Additionally, when xenografts show intense staining of vWF, EC activation is indicated, which is a precursor to coagulation‐related complications.^47^ Controlling this activation is essential to avoiding thrombotic complications post‐transplant. However, pigs with humanized vWF have not yet played a role in pig‐to‐NHP kidney transplantation.

### 5.2 Expression of human coagulation‐regulatory proteins

To prevent accelerated clotting, TFPI expression has been introduced into pig ECs, thereby addressing the incompatibility between pig TFPI and human TF, which contributes to coagulation problems.^40^ This incompatibility may stem from the α isoform (TFPIα), as the inhibition of human TF by pig TFPIα was found to be inefficient.^13^ Moreover, porcine TFPI fails to neutralize human FXa and there is aberrant activation of both human prothrombin and factor X by porcine ECs in vitro and ex vivo.^48^

Pig ECs that have been genetically modified to express human thrombomodulin show promise in reducing prothrombinase activity, thereby inhibiting coagulation.^9^ This modification, coupled with the introduction of hEPCR, enhances the activation of protein C, forming a comprehensive strategy to mitigate coagulation‐related complications in xenotransplantation.^9^ Additionally, human thrombomodulin and ectonucleoside triphosphate diphosphohydrolase‐1 (CD39) play crucial roles in modulating inflammation and coagulation.^28^, ^49^ CD39, in particular, helps prevent thrombosis by breaking down platelet agonists.^28^, ^50^ Overexpression of human CD39 (hCD39) in pigs has been found effective in reducing clot formation.^51^, ^52^ To achieve prolonged xenograft survival, it has been suggested that the CD39/vascular ATP diphosphohydrolase interaction modulates xenograft survival.^52^ In addition, human thrombomodulin modifies thrombin specificity, offering protection against coagulation during xenotransplantation.^25^ This is evidenced by research showing that baboons receiving hearts from pigs transgenic for human thrombomodulin did not exhibit thrombocytopenia or decreased fibrinogen levels, indicating a lower incidence of coagulation disorders.^53^ In addition, when comparing kidney and heart xenotransplantation to liver xenotransplantation, expression of various human coagulation proteins have been associated with differing outcomes. Namely, excessive platelet sequestration soon after liver xenotransplantation has been associated with rapid thrombocytopenia independent of organ rejection, a feature that is not typical for heart or kidney xenotransplantation.^53^ Moreover, other genetic modifications in pigs, such as combining GTKO with anti‐inflammatory proteins like HO‐1 and/or A20, have shown potential in protecting pig kidneys from rejection/inflammation during ex vivo perfusion with human blood.^32^

When hEPCR was *not* included in genetic modifications of xenografts, graft survival significantly decreased and CC was observed.^54^ This is in comparison with survival of >7 months with the inclusion of hEPCR, suggesting that hEPCR in the graft is critically important to avoid coagulation dysregulation.^54^ However, different effects on complement activation and subsequent coagulation dysregulation have been observed in xenografts expressing hCD55 or hEPCR.^55^

Expression of hCD47 on pig cells not only suppresses the activation of human macrophages and inflammatory cytokine production^8^ but also is implicated in the inhibition of platelets.^8^ A summary of these select genetic modifications can be discerned in Table 4.

**TABLE 4:** Common graft genetic modifications with anticoagulation and/or antiplatelet effects.^20^, ^22^, ^26^, ^27^, ^28^, ^37^, ^40^, ^48^, ^50^, ^52^

Genetic modification	In vitro	In vivo
Thrombomodulin (TBM)^20^, ^22^	Accelerates thrombin‐mediated PC activation; inhibits thrombin‐induced platelet aggregation; reduces prothrombinase activity	Prolonged survival in xenotransplant models; reduction in consumptive coagulopathy occurrence
Endothelial cell protein C receptor (EPCR)^26^, ^27^, ^37^	Reduced thrombin generation; decreased endothelial cell apoptosis; reduction in platelet aggregation activity	Reduction in thrombus formation; extends survival time in xenotransplant models
CD39^28^, ^50^, ^52^	Decreased platelet aggregation; reduced P‐selectin expression on platelets	Associated with preventing clotting in NHP models; significantly prolongs graft survival
Tissue factor pathway inhibitor (TFPI)^40^, ^48^	Reduced thrombin formation; decreased fibrin deposition	Reduction in clot formation; improved graft perfusion

## 6 EFFECTS OF SELECTED DRUGS ON COAGULATION DYSREGULATION IN XENOTRANSPLANTATION

Drugs administered in kidney xenotransplantation may be potentially beneficial or detrimental in controlling coagulation dysregulation. At present, although the innate immune response can largely be prevented by gene‐editing of the pig, it remains essential to administer immunosuppressive therapy to prevent the adaptive immune response. The two main classes of drugs that must be considered are therefore immunosuppressants and anticoagulant/antiplatelet agents. As a result of >25 years' experience in pig‐to‐NHP models of xenotransplantation, most immunosuppressive regimens today are based on blockade of the CD40/CD154 T‐cell costimulation pathway.

### 6.1 CD40/CD154 costimulation pathway blockade

Anti‐CD154 mAb was originally employed very successfully in NHP studies as a means to improve long‐term graft survival, but its use was limited because of its thrombogenic properties.^30^, ^56^, ^57^ In an attempt to prevent thrombotic complications, anti‐CD154 mAb was administered with ketorolac 1 mg/kg but was not extensively used.^58^, ^59^, ^60^ It was replaced by anti‐CD40mAb which, although not thrombogenic, was less effective. Recent novel anti‐CD154 domain antibodies have been developed to reduce potential thromboembolism by preventing platelet activation.^61^ Several anti‐CD40 and anti‐CD154 agents targeting the CD40/CD40L pathway are in the pipeline, including ch5D12, 3A8(3A8R1), Chi220, 2C10R4, Bleselumab, Iscalimab, KPL‐404, tegoprubart, and TNX‐1500.^62^ Several are showing encouraging results in models of allotransplantation and xenotransplantation.^27^, ^63^, ^64^, ^65^ However, neither anti‐CD40mAbs nor anti‐CD154mAbs have yet been approved by the US Food and Drug Administration (FDA) as therapy for organ transplantation.^32^, ^58^

### 6.2 Other immunosuppressive agents

The calcineurin inhibitors, for example, tacrolimus, have not been demonstrated to be as effective in preventing rejection of xenografts as agents that block the CD40/CD154 costimulation pathway.^56^, ^66^ Rapamycin is an effective immunosuppressant, but there is some evidence suggesting that it promotes thrombosis (via reduced tPA expression and inducing PAI‐1/TF expression)^67^ and TMA^28^ (Table 5). However, when combined with an anti‐CD154mAb in the pig‐to‐NHP kidney transplant model, rapamycin has not been noted to promote thrombosis.^65^

**TABLE 5:** Selected anticoagulant and immunosuppressive drugs administered after pig‐to‐NHP kidney xenotransplantation.^72^, ^73^, ^74^, ^75^, ^76^, ^77^, ^78^, ^79^, ^80^, ^81^

Drug	Action	Advantages	Limitations	Results (in vitro, in vivo)
Recombinant human‐activated protein C (rhAPC)^72^	Anticoagulant	More effective in preventing thrombin activity and fibrin deposition compared with rhAT^72^	Increased risk of bleeding with high doses^73^ Limited clinical data supporting use in kidney transplantation^72^	Animals treated with rhAPC survived between 8 and 55 days post‐transplantation rhAPC inhibited thrombin generation and F1 + 2 generation in a concentration‐dependent manner^72^
Recombinant human antithrombin (rhAT)^74^	Anticoagulant	At low doses, prevents thrombotic events in the first week post‐xenotransplant at low doses^74^	Implicated with bleeding following surgery^75^	rhAT was effective in delaying the onset of rejection and preventing coagulopathy in pig‐to‐primate renal xenotransplantation and maintained renal function twice as long as untreated animals^74^
Heparin^77^	Anticoagulant	Improves graft function by reducing clot formation^76^ Reduction in ischemia–reperfusion injury^77^	Heparin‐induced thrombocytopenia^78^ Increased risk of bleeding Implicated with hemorrhage in xenotransplantation.	Heparin reduced ischemia–reperfusion injury in end organs such as the lungs and heart in animal models, decreasing levels of inflammatory markers like myeloperoxidase and heat shock protein 70^77^
Tocilizumab^79^	Anti‐inflammatory (IL‐6R—interleukin 6 receptor)	Protective effect on renal function^79^ Reduces inflammation^79^	High infection rate^79^	Clinical trials show Tocilizumab can be an effective treatment for antibody‐mediated rejection in kidney transplant patients^79^
Rapamycin^80^	Immuno‐suppressant	Suppresses platelet procoagulant responses by protecting mitochondrial integrity^80^	Potentially causes delayed wound healing^81^	Rapamycin restrains platelet procoagulant responses via FKBP‐mediated protection of mitochondrial integrity, maintaining mitochondrial potential and reducing procoagulant activity of platelets^80^

The “anti‐inflammatory” agent, tocilizumab, that targets the IL‐6 receptor, may mitigate antibody‐mediated rejection, consumptive coagulation, and other coagulation hurdles in pig‐to‐NHP transplants.^68^ The combination of an anti‐CD154mAb, rapamycin, and tocilizumab is providing successful results.^65^

### 6.3 Anticoagulants

**FIGURE 3:**
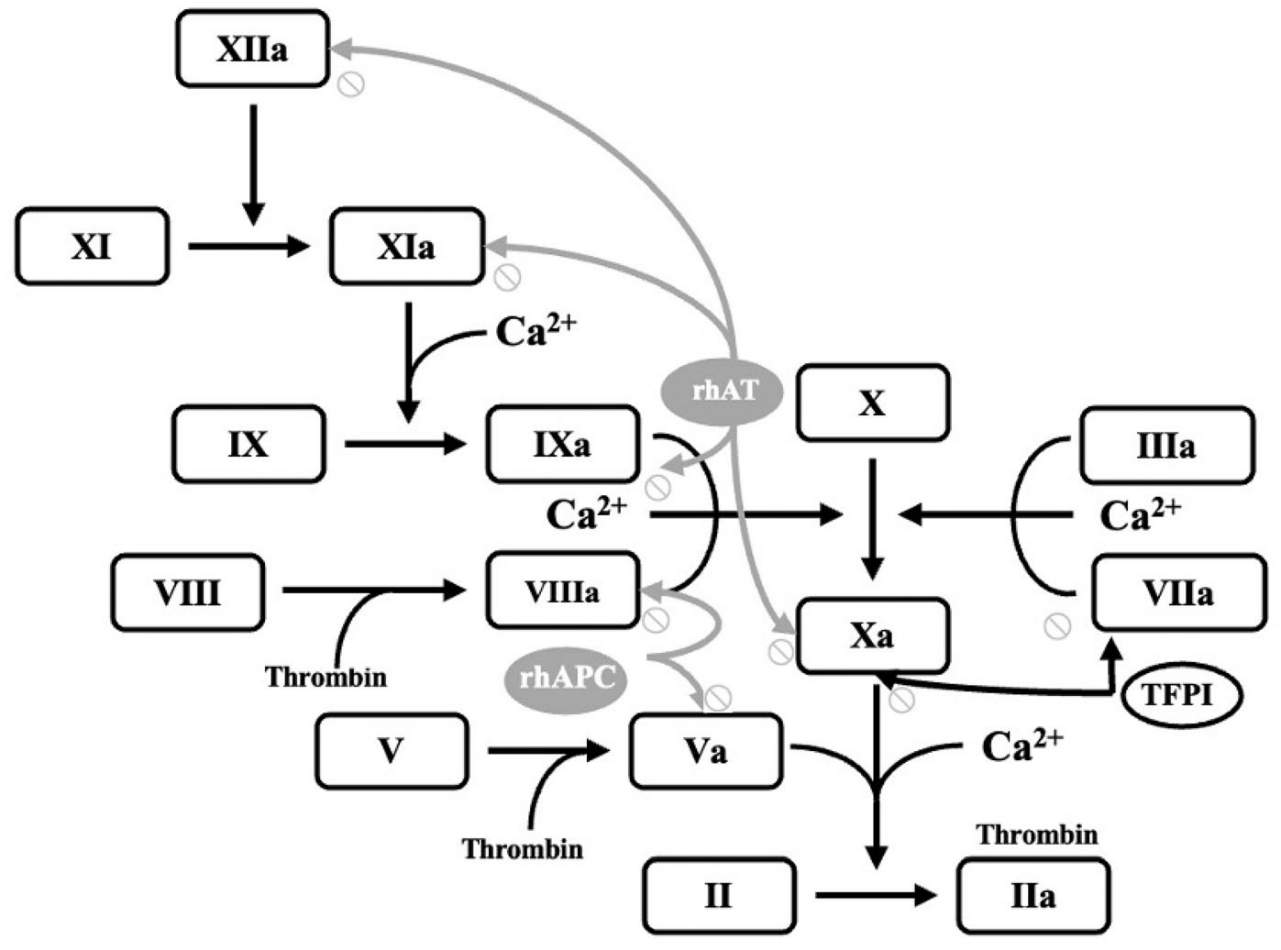
Mechanisms of rhAT and rhAPC in overcoming coagulation dysregulation in pig kidney xenotransplantation. RhAT and rhAPC are shown inhibiting key steps in the cascade, particularly the activities of Factor Xa and Va, thereby regulating thrombin generation and preventing excessive clot formation. RhAT, recombinant human antithrombin; rhAPC, recombinant human‐activated protein C.

Most work aimed at preventing coagulation dysregulation in xenotransplantation has been directed to gene‐editing of the pig, but the effect of a few anticoagulants has been investigated. Recombinant human‐activated protein C (rhAPC) is a synthetic form of activated protein C which functions by inhibiting clot formation through multiple pathways, primarily by degrading FVa and FVIIIa, thereby downregulating the clotting cascade (Figure 3).^69^ rhAPC in combination with recombinant human antithrombin (rhAT) led to the survival of a hDAF/Gal^+^ pig organ for 34 days.^19^ rhAT operates as a natural coagulation inhibitor by targeting several factors within the clotting cascade, specifically by neutralizing thrombin and factors IXa, Xa, XIa, and XIIa (Figure 3).^70^ Its mechanism involves binding to these coagulation factors, which ultimately impedes the conversion of fibrinogen to fibrin and interrupts clot formation.^70^ In an ex vivo wild‐type pig‐to‐human kidney model, Ramackers et al. found that high doses of rhAT can mitigate xenogeneic activation of hemostasis and prevent TMA by reducing the activation of platelets and endothelial cells. This is evidenced by reducing the increase in D‐dimer and fibrinogen consumption.^69^ Similar results were observed using rhAPC.^69^ However, concern has been raised about possible bleeding complications associated with the perioperative use of these agents.^69^

Heparin has also been used in kidney xenotransplantation models in NHPs and in brain‐dead humans. One decedent with a pig kidney graft developed exsanguinating hemorrhage from severe coagulopathy, but this may have been associated with the effects of brain death.^71^

## 7 CONCLUSIONS

Cumulatively, the shortage of human kidneys has catalyzed the exploration of kidney xenotransplantation to address the dire need for transplantable organs and bypass blood type compatibility issues. Despite its promise, managing coagulation dysregulation remains a pivotal concern among high rates of antibody‐mediated rejection, physiological incompatibilities, acute cellular rejection, and the risk of infection between species. The complexity of coagulation, involving intricate interactions between various factors, is further complicated in the xenotransplantation context, particularly by abnormal pig vWF interactions, inefficient pig thrombomodulin, thrombotic microangiopathy, and consumptive coagulopathy. Innovative solutions, such as genetic engineering and the utility of adequate immunosuppressive and anticoagulant drugs, have shown promise in overcoming these hurdles but warrant further investigation.

## AUTHOR CONTRIBUTIONS

Ali Zidan contributed to the study's conceptualization and design. All authors significantly contributed to the writing, drafting, and editing of the manuscript. All authors have read and approved the manuscript for submission to the American Journal of Hematology.

## CONFLICT OF INTEREST STATEMENT

DKCC is a consultant to eGenesis Bio of Cambridge, MA, but the opinions expressed in this article are those of the authors and do not necessarily reflect those of eGenesis. The other authors indicate no conflicts of interest.

## PATIENT CONSENT STATEMENT

No patient consent was required for the preparation of this review article, as it did not involve the collection of new data from patients or direct interactions with any individuals. The data analyzed were derived from previously published research articles where the necessary patient consents had been obtained by the original researchers, as detailed in their respective publications.

## Data Availability

Data sharing not applicable to this article as no datasets were generated or analyzed during the current study.

## References

[1] Kovesdy CP . Epidemiology of chronic kidney disease: an update 2022. Kidney Int Suppl. 2022;12(1):7‐11. doi:10.1016/j.kisu.2021.11.003 PMC9073222 35529086

[2] Organ Procurement and Transplantation Network . Current U.S. waiting list. 2024 Accessed March 20, 2024. https://optn.transplant.hrsa.gov/data/view-data-reports/national-data/

[3] Mudiayi D , Shojai S , Okpechi I , et al. Global estimates of capacity for kidney transplantation in world countries and regions. Transplantation. 2022;106(6):1113‐1122. doi:10.1097/TP.0000000000003943 34495014 PMC9128615

[4] Husain SA , Brennan C , Michelson A , et al. Patients prioritize waitlist over posttransplant outcomes when evaluating kidney transplant centers. Am J Transplant. 2018;18(11):2781‐2790. doi:10.1111/ajt.14985 29945305 PMC6314030

[5] Cooper DKC , Pierson RN . Milestones on the path to clinical pig organ xenotransplantation. Am J Transplant. 2023;23(3):326‐335. doi:10.1016/j.ajt.2022.12.023 36775767 PMC10127379

[6] Cooper DKC , Iwase H , Wang L , et al. Bringing home the bacon: update on the state of kidney xenotransplantation. Blood Purif. 2018;45(1–3):254‐259. doi:10.1159/000485163 29478054

[7] Firl DJ , Markmann JF . Measuring success in pig to non‐human‐primate renal xenotransplantation: systematic review and comparative outcomes analysis of 1051 life‐sustaining NHP renal allo‐ and xeno‐transplants. Am J Transplant. 2022;22(6):1527‐1536. doi:10.1111/ajt.16994 35143091

[8] Zhang X , Wang Q , Zhao J , et al. The resurgent landscape of xenotransplantation of pig organs in nonhuman primates. Sci China Life Sci. 2021;64(5):697‐708. doi:10.1007/s11427-019-1806-2 32975720

[9] Cowan PJ , Robson SC , d'Apice AJ . Controlling coagulation dysregulation in xenotransplantation. Curr Opin Organ Transplant. 2011;16(2):214‐221. doi:10.1097/MOT.0b013e3283446c65 21415824 PMC3094512

[10] Chase B . World's first genetically‐edited pig kidney transplant into living recipient performed at Massachusetts General Hospital. 2024 Accessed April 1, 2024. https://www.massgeneral.org/news/press-release/worlds-first-genetically-edited-pig-kidney-transplant-into-living-recipient

[11] Sang Y , Roest M , de Laat B , de Groot PG , Huskens D . Interplay between platelets and coagulation. Blood Rev. 2021;46:100733. doi:10.1016/j.blre.2020.100733 32682574 PMC7354275

[12] Al‐Koussa H , AlZaim I , El‐Sabban ME . Pathophysiology of coagulation and emerging roles for extracellular vesicles in coagulation cascades and disorders. J Clin Med. 2022;11(16):4932. doi:10.3390/jcm11164932 36013171 PMC9410115

[13] Kopp C , Siegel J , Hancock W , et al. Effect of porcine endothelial tissue factor pathway inhibitor on human coagulation factors. Transplantation. 1997;63(5):749‐758. 9075849 10.1097/00007890-199703150-00023

[14] Ji H , Li X , Yue S , et al. Pig BMSCs transfected with human TFPI combat species incompatibility and regulate the human TF pathway in vitro and in a rodent model. Cell Phys Biochem. 2015;36(1):233‐249. doi:10.1159/000374067 25967963

[15] Lin H , Xu L , Yu S , Hong W , Huang M , Xu P . Therapeutics targeting the fibrinolytic system. Exp Mol Med. 2020;52(3):367‐379. doi:10.1038/s12276-020-0397-x 32152451 PMC7156416

[16] Connolly MR , Kuravi K , Burdorf L , et al. Humanized von Willebrand factor reduces platelet sequestration in ex vivo and in vivo xenotransplant models. Xenotransplantation. 2021;28(6):e12712. doi:10.1111/xen.12712 34657336 PMC10266522

[17] Loupy A , Goutaudier V , Giarraputo A , et al. Immune response after pig‐to‐human kidney xenotransplantation: a multimodal phenotyping study. Lancet. 2023;402(10408):1158‐1169. doi:10.1016/S0140-6736(23)01349-1 37598688

[18] Cooper DKC , Gaston R , Eckhoff D , et al. Xenotransplantation—the current status and prospects. Br Med Bull. 2018;125(1):5‐14. doi:10.1093/bmb/ldx043 29228112 PMC6487536

[19] Spiezia L , Boldrin M , Radu C , et al. Thromboelastographic evaluation of coagulative profiles in pig‐to‐monkey kidney xenotransplantation. Xenotransplantation. 2013;20(2):89‐99. doi:10.1111/xen.12024 23406330

[20] Roussel JC , Moran CJ , Salvaris EJ , Nandurkar HH , D'Apice AJF , Cowan PJ . Pig thrombomodulin binds human thrombin but is a poor cofactor for activation of human protein C and TAFI. Am J Transplant. 2008;8(6):1101‐1112. doi:10.1111/j.1600-6143.2008.02210.x 18444940

[21] Siegel J , Grey S , Lesnikoski BA , et al. Xenogeneic endothelial cells activate human prothrombin. Transplantation. 1997;64(6):888‐896. 9326416 10.1097/00007890-199709270-00017

[22] Hara H , Iwase H , Nguyen H , et al. Stable expression of the human thrombomodulin transgene in pig endothelial cells is associated with a reduction in the inflammatory response. Cytokine. 2021;148:155580. doi:10.1016/j.cyto.2021.155580 34099346 PMC8511266

[23] Kopp C , Grey S , Siegel J , et al. Expression of human thrombomodulin cofactor activity in porcine endothelial cells. Transplantation. 1998;66(2):244‐251. 9701273 10.1097/00007890-199807270-00019

[24] Mohiuddin MM , Singh AK , Corcoran PC , et al. Chimeric 2C10R4 anti‐CD40 antibody therapy is critical for long‐term survival of GTKO.hCD46.hTBM pig‐to‐primate cardiac xenograft. Nat Commun. 2016;7(1):11138. doi:10.1038/ncomms11138 27045379 PMC4822024

[25] Iwase H , Ekser B , Hara H , et al. Regulation of human platelet aggregation by genetically modified pig endothelial cells and thrombin inhibition. Xenotransplantation. 2014;21(1):72‐83. doi:10.1111/xen.12073 24188473 PMC4010578

[26] Mohan Rao LV , Esmon CT , Pendurthi UR . Endothelial cell protein C receptor: a multiliganded and multifunctional receptor. Blood. 2014;124(10):1553‐1562. doi:10.1182/blood-2014-05-578328 25049281 PMC4155268

[27] Anand RP , Layer JV , Heja D , et al. Design and testing of a humanized porcine donor for xenotransplantation. Nature. 2023;622(7982):393‐401. doi:10.1038/s41586-023-06594-4 37821590 PMC10567564

[28] Tector AJ , Adams AB , Tector M . Current status of renal xenotransplantation and next steps. Kidney360. 2023;4(2):278‐284. doi:10.34067/KID.0007152021 36821619 PMC10103350

[29] Sykes M , Sachs DH . Progress in xenotransplantation: overcoming immune barriers. Nat Rev Nephrol. 2022;18(12):745‐761. doi:10.1038/s41581-022-00624-6 36198911 PMC9671854

[30] Wijkstrom M , Iwase H , Paris W , Hara H , Ezzelarab M , Cooper DKC . Renal xenotransplantation: experimental progress and clinical prospects. Kidney Int. 2017;91(4):790‐796. doi:10.1016/j.kint.2016.08.035 27914702 PMC5357451

[31] Peri S , Kulkarni A , Feyertag F , Berninsone PM , Alvarez‐Ponce D . Phylogenetic distribution of CMP‐Neu5Ac hydroxylase (CMAH), the enzyme synthetizing the proinflammatory human xenoantigen Neu5Gc. Genome Biol Evol. 2018;10(1):207‐219. doi:10.1093/gbe/evx251 29206915 PMC5767959

[32] Yu XH , Deng WY , Jiang HT , Li T , Wang Y . Kidney xenotransplantation: recent progress in preclinical research. Clin Chim Acta. 2021;514:15‐23. doi:10.1016/j.cca.2020.11.028 33301767

[33] Ghanekar A , Mendicino M , Liu H , et al. Endothelial induction of fgl2 contributes to thrombosis during acute vascular xenograft rejection. J Immun. 2004;172(9):5693‐5701. doi:10.4049/jimmunol.172.9.5693 15100314

[34] Müller F , Mutch NJ , Schenk WA , et al. Platelet polyphosphates are proinflammatory and procoagulant mediators in vivo. Cell. 2009;139(6):1143‐1156. doi:10.1016/j.cell.2009.11.001 20005807 PMC2796262

[35] Salvaris EJ , Moran CJ , Roussel JC , Fisicaro N , Robson SC , Cowan PJ . Pig endothelial protein C receptor is functionally compatible with the human protein C pathway. Xenotransplantation. 2020;27(2):e12557. doi:10.1111/xen.12557 31556182

[36] Montgomery RA , Stern JM , Lonze BE , et al. Results of two cases of pig‐to‐human kidney xenotransplantation. N Engl J Med. 2022;386(20):1889‐1898. doi:10.1056/NEJMoa2120238 35584156

[37] Cowan PJ , Cooper DKC , d'Apice AJF . Kidney xenotransplantation. Kidney Int. 2014;85(2):265‐275. doi:10.1038/ki.2013.381 24088952 PMC3946635

[38] Zhou H , Hara H , Cooper DKC . The complex functioning of the complement system in xenotransplantation. Xenotransplantation. 2019;26(4):e12517. doi:10.1111/xen.12517 31033064 PMC6717021

[39] Lee KFE , Lu B , Roussel JC , et al. Protective effects of transgenic human endothelial protein c receptor expression in murine models of transplantation. Am J Transplant. 2012;12(9):2363‐2372. doi:10.1111/j.1600-6143.2012.04122.x 22681753

[40] Lu T , Yang B , Wang R , Qin C . Xenotransplantation: current status in preclinical research. Front Immunol. 2020;10:3060. doi:10.3389/fimmu.2019.03060 32038617 PMC6989439

[41] Cooper DKC , Hara H . “You cannot stay in the laboratory forever”*: taking pig kidney xenotransplantation from the laboratory to the clinic. EBioMedicine. 2021;71:103562. doi:10.1016/j.ebiom.2021.103562 34517284 PMC8441149

[42] Wang L , Cooper DKC , Burdorf L , Wang Y , Iwase H . Overcoming coagulation dysregulation in pig solid organ transplantation in nonhuman primates. Transplantation. 2018;102(7):1050‐1058. doi:10.1097/TP.0000000000002171 29538262 PMC7228622

[43] Lin CC , Ezzelarab M , Shapiro R , et al. Recipient tissue factor expression is associated with consumptive coagulopathy in pig‐to‐primate kidney xenotransplantation. Am J Transplant. 2010;10(7):1556‐1568. doi:10.1111/j.1600-6143.2010.03147.x 20642682 PMC2914318

[44] Eisenson DL , Hisadome Y , Yamada K . Progress in xenotransplantation: immunologic barriers, advances in gene editing, and successful tolerance induction strategies in pig‐to‐primate transplantation. Front Immunol. 2022;13:899657. doi:10.3389/fimmu.2022.899657 35663933 PMC9157571

[45] Cantu E , Balsara K , Li B , et al. Prolonged function of macrophage, von willebrand factor‐deficient porcine pulmonary xenografts. Am J Transplant. 2007;7(1):66‐75. doi:10.1111/j.1600-6143.2006.01603.x 17109734

[46] Schulte am Esch J , Robson SC , Knoefel WT , Hosch SB , Rogiers X . O‐linked glycosylation and functional incompatibility of porcine von Willebrand factor for human platelet GPIb receptors. Xenotransplantation. 2005;12(1):30‐37. doi:10.1111/j.1399-3089.2004.00187.x 15598271

[47] Kalagara T , Moutsis T , Yang Y , et al. The endothelial glycocalyx anchors von Willebrand factor fibers to the vascular endothelium. Blood Adv. 2018;2(18):2347‐2357. doi:10.1182/bloodadvances.2017013995 30237293 PMC6156889

[48] Robson S , Esch JS , Bach F . Factors in cenograft rejection. Ann N Y Acad Sci. 1999;875(1):261‐276. doi:10.1111/j.1749-6632.1999.tb08509.x 10415573

[49] Kim SC , Mathews DV , Breeden CP , et al. Long‐term survival of pig‐to‐rhesus macaque renal xenografts is dependent on CD4 T cell depletion. Am J Transplant. 2019;19(8):2174‐2185. doi:10.1111/ajt.15329 30821922 PMC6658347

[50] Kaczmarek E , Koziak K , Sévigny J , et al. Identification and characterization of CD39/vascular ATP diphosphohydrolase. J Biol Chem. 1996;271(51):33116‐33122. doi:10.1074/jbc.271.51.33116 8955160

[51] Crikis S , Lu B , Murray‐Segal LM , et al. Transgenic overexpression of CD39 protects against renal ischemia‐reperfusion and transplant vascular injury. Am J Transplant. 2010;10(12):2586‐2595. doi:10.1111/j.1600-6143.2010.03257.x 20840479 PMC5472986

[52] Imai M , Takigami K , Guckelberger O , et al. Recombinant adenoviral mediated CD39 gene transfer prolongs cardiac xenograft survival. Transplantation. 2000;70(6):864‐870. doi:10.1097/00007890-200009270-00003 11014639

[53] Iwase H , Ekser B , Satyananda V , et al. Pig‐to‐baboon heterotopic heart transplantation – exploratory preliminary experience with pigs transgenic for human thrombomodulin and comparison of three costimulation blockade‐based regimens. Xenotransplantation. 2015;22(3):211‐220. doi:10.1111/xen.12167 25847282 PMC4464944

[54] Iwase H , Hara H , Ezzelarab M , et al. Immunological and physiological observations in baboons with life‐supporting genetically engineered pig kidney grafts. Xenotransplantation. 2017;24(2):12293. doi:10.1111/xen.12293 PMC5397334 28303661

[55] Harris DG , Quinn KJ , French BM , et al. Meta‐analysis of the independent and cumulative effects of multiple genetic modifications on pig lung xenograft performance during ex vivo perfusion with human blood. Xenotransplantation. 2015;22(2):102‐111. doi:10.1111/xen.12149 25470239 PMC4390422

[56] Bühler L , Awwad M , Basker M , et al. High‐dose porcine hematopoietic cell transplantation combined with CD40 ligand blockade in baboons prevents an induced anti‐pig humoral response. Transplantation. 2000;69(11):2296‐2304. doi:10.1097/00007890-200006150-00013 10868629

[57] Samy KP , Butler JR , Li P , Cooper DKC , Ekser B . Corrigendum to “the role of costimulation blockade in solid organ and islet xenotransplantation”. J Immunol Res. 2018;2018:1‐2. doi:10.1155/2018/6343608 PMC5885337 29767012

[58] Ma D , Hirose T , Lassiter G , et al. Kidney transplantation from triple‐knockout pigs expressing multiple human proteins in cynomolgus macaques. Am J Transplant. 2022;22(1):46‐57. doi:10.1111/ajt.16780 34331749 PMC9291868

[59] Kawai T , Andrews D , Colvin RB , Sachs DH , Cosimi AB . Thromboembolic complications after treatment with monoclonal antibody against CD40 ligand. Nat Med. 2000;6(2):114. doi:10.1038/72162 10655073

[60] Knosalla C , Gollackner B , Cooper DKC . Anti‐CD154 monoclonal antibody and thromboembolism revisited. Transplantation. 2002;74(3):416‐417. doi:10.1097/00007890-200208150-00024 12184326

[61] Kim SC , Wakwe W , Higginbotham LB , et al. Fc‐silent anti‐CD154 domain antibody effectively prevents nonhuman primate renal allograft rejection. Am J Transplant. 2017;17(5):1182‐1192. doi:10.1111/ajt.14197 28097811 PMC5409881

[62] Singh AK , Goerlich CE , Zhang T , Lewis BGT , Hershfeld A , Mohiuddin MM . CD40‐CD40L blockade: update on novel investigational therapeutics for transplantation. Transplantation. 2023;107(7):1472‐1481. doi:10.1097/TP.0000000000004469 36584382 PMC10287837

[63] Kitchens WH , Larsen CP , Badell IR . Costimulatory blockade and solid organ transplantation: the past, present, and future. Kidney Int Rep. 2023;8(12):2529‐2545. doi:10.1016/j.ekir.2023.08.037 38106575 PMC10719580

[64] Perrin S , Magill M . The inhibition of CD40/CD154 costimulatory signaling in the prevention of renal transplant rejection in nonhuman primates: a systematic review and meta analysis. Front Immunol. 2022;13:861471. doi:10.3389/fimmu.2022.861471 35464470 PMC9022482

[65] Kinoshita K , Maenaka A , Rosales I , et al. Novel factors potentially initiating acute antibody‐mediated rejection in pig kidney xenografts despite an efficient immunosuppressive Regimen. Xenotransplantation. 2024;31:e12859. 38646924 10.1111/xen.12859 PMC13007565

[66] Yamamoto T , Hara H , Foote J , et al. Life‐supporting kidney xenotransplantation from genetically engineered pigs in baboons: a comparison of two immunosuppressive regimens. Transplantation. 2019;103(10):2090‐2104. doi:10.1097/TP.0000000000002796 31283686

[67] Jiang P , Lan Y , Luo J , et al. Rapamycin promoted thrombosis and platelet adhesion to endothelial cells by inducing membrane remodeling. BMC Cell Biol. 2014;15(1):7. doi:10.1186/1471-2121-15-7 24564184 PMC3936831

[68] Li T , Hara H , Ezzelarab MB , et al. Serum amyloid a as a marker of inflammation in xenotransplantation. Eur J Inflamm. 2018;16:205873921878004. doi:10.1177/2058739218780046

[69] Ramackers W , Friedrich L , Klose J , et al. Recombinant human antithrombin prevents xenogenic activation of hemostasis in a model of pig‐to‐human kidney transplantation. Xenotransplantation. 2014;21(4):367‐375. doi:10.1111/xen.12104 24807299

[70] Palta S , Saroa R , Palta A . Overview of the coagulation system. Indian J Anaesth. 2014;58(5):515‐523. doi:10.4103/0019-5049.144643 25535411 PMC4260295

[71] Porrett PM , Orandi BJ , Kumar V , et al. First clinical‐grade porcine kidney xenotransplant using a human decedent model. Am J Transplant. 2022;22(4):1037‐1053. doi:10.1111/ajt.16930 35049121

[72] Simioni P , Boldrin M , Gavasso S , et al. Effects of long‐term administration of recombinant human protein C in xenografted primates. Transplantation. 2011;91(2):161‐168. doi:10.1097/TP.0b013e318200ba0e 21088649

[73] Tanaka K , Tawara S , Tsuruta K , Hoppensteadt D , Fareed J . Pharmacological differentiation of thrombomodulin alfa and activated protein C on coagulation and fibrinolysis in vitro. Clin Appl Thromb Hemost. 2018;24(6):859‐866. doi:10.1177/1076029618770274 29683037 PMC6714727

[74] Cowan PJ , Aminian A , Barlow H , et al. Protective effects of recombinant human antithrombin III in pig‐to‐primate renal xenotransplantation. Am J Transplantation. 2002;2(6):520‐525. doi:10.1034/j.1600-6143.2002.20605.x 12118895

[75] Avidan MS , Levy JH , Scholz J , et al. A phase III, double‐blind, placebo‐controlled, multicenter study on the efficacy of recombinant human antithrombin in heparin‐resistant patients scheduled to undergo cardiac surgery necessitating cardiopulmonary bypass. Anesthesiology. 2005;102(2):276‐284. doi:10.1097/00000542-200502000-00007 15681940

[76] Surianarayanan V , Hoather TJ , Tingle SJ , Thompson ER , Hanley J , Wilson CH . Interventions for preventing thrombosis in solid organ transplant recipients. Cochrane Database Syst Rev. 2021;2021(3):CD011557. doi:10.1002/14651858.CD011557.pub2 PMC8094924 33720396

[77] Gedik HS , Korkmaz K , Erdem H , Karakilic E , Lafci G , Ankarali H . Protective effect of heparin in the end organ ischemia/reperfusion injury of the lungs and heart. J Cardiothorac Surg. 2012;7(1):123. doi:10.1186/1749-8090-7-123 23151309 PMC3558397

[78] Arepally GM , Padmanabhan A . Heparin‐induced thrombocytopenia. Arterioscler Thromb Vasc Biol. 2020;41:141‐152. doi:10.1161/ATVBAHA.120.315445 33267665 PMC7769912

[79] Cabezas L , Jouve T , Malvezzi P , et al. Tocilizumab and active antibody‐mediated rejection in kidney transplantation: a literature review. Front Immunol. 2022;13:839380. doi:10.3389/fimmu.2022.839380 35493469 PMC9047937

[80] Śledź KM , Moore SF , Durrant TN , Blair TA , Hunter RW , Hers I . Rapamycin restrains platelet procoagulant responses via FKBP‐mediated protection of mitochondrial integrity. Biochem Pharmacol. 2020;177:113975. doi:10.1016/j.bcp.2020.113975 32298692

[81] Mabood Khalil MA , Al‐Ghamdi SMG , Dawood US , et al. Mammalian target of rapamycin inhibitors and wound healing complications in kidney transplantation: old myths and new realities. J Transplant. 2022;2022:1‐28. doi:10.1155/2022/6255339 PMC8901320 35265364

